# A Web Server for Designing Molecular Switches Composed of Two Interacting RNAs

**DOI:** 10.3390/ijms22052720

**Published:** 2021-03-08

**Authors:** Akito Taneda, Kengo Sato

**Affiliations:** 1 Graduate School of Science and Technology, Hirosaki University, Hirosaki, Aomori 036-8561, Japan; 2 Department of Biosciences and Informatics, Keio University, 3-14-1 Hiyoshi, Kohoku-ku, Yokohama 223-8522, Japan; satoken@bio.keio.ac.jp

**Keywords:** secondary structure, RNA–RNA interaction, in silico design, synthetic biology, genetic algorithm, multi-objective optimization

## Abstract

The programmability of RNA–RNA interactions through intermolecular base-pairing has been successfully exploited to design a variety of RNA devices that artificially regulate gene expression. An in silico design for interacting structured RNA sequences that satisfies multiple design criteria becomes a complex multi-objective problem. Although multi-objective optimization is a powerful technique that explores a vast solution space without empirical weights between design objectives, to date, no web service for multi-objective design of RNA switches that utilizes RNA–RNA interaction has been proposed. We developed a web server, which is based on a multi-objective design algorithm called MODENA, to design two interacting RNAs that form a complex in silico. By predicting the secondary structures with RactIP during the design process, we can design RNAs that form a joint secondary structure with an external pseudoknot. The energy barrier upon the complex formation is modeled by an interaction seed that is optimized in the design algorithm. We benchmarked the RNA switch design approaches (MODENA+RactIP and MODENA+RNAcofold) for the target structures based on natural RNA-RNA interactions. As a result, MODENA+RactIP showed high design performance for the benchmark datasets.

## 1. Introduction

Synthetic RNA molecules are promising biomolecular devices that provide powerful means for artificially controlling gene expression [1,2,3,4]. To date, various synthetic functional RNAs have been developed based on the modularity of RNA; i.e., a number of synthetic RNA molecules have been designed by combining functional components, such as ribozymes, aptamers, and conformational changes. In such attractive features of RNA, the programmability of RNA–RNA interactions through intermolecular base-pair formation is particularly useful in designing functional RNA sequences that specifically bind to a targeted locus. By exploiting this advantage of RNA molecules, various RNA–RNA switches (i.e., RNA switches utilizing RNA–RNA interaction) that have characteristic functional secondary structures have been developed to artificially regulate gene expression. Small transcription-activating RNAs (STARs), which are antisense RNAs that bind and prevent the stem-loop structure of a transcription terminator, control gene expression [5]. In toehold switches [6], a trigger RNA specifically binds to a 5’UTR region that has a stem structure that sequesters ribosome binding site (RBS); this binding opens the stem structure of the RBS region, leading to expression of the downstream gene. Clustered Regularly Interspaced Short Palindromic Repeat (CRISPR)-based regulation relies on small guide RNAs (sgRNAs) so that CRISPR guide riboswitches are engineered by designing an sgRNA that responds to a trigger RNA [7,8]. Ultimately, these RNA–RNA switches can be integrated in order to construct artificial genetic circuits [2,9,10]. Thus, interacting structured RNAs are attractive designable biomolecules for synthetic biology.

RNA inverse folding, which automatically explores an RNA sequence space to find RNA sequences that fold into a specified target structure, has been utilized to obtain RNA sequences that have desired structural features and are functional in vitro/in vivo [9,11,12]. To date, a number of RNA inverse folding algorithms for single-sequence design have been proposed (for a review, see [13]). In contrast to the plentifulness of the single-sequence inverse folding methods, inverse folding tools for interacting RNAs (particularly for RNA–RNA switches) are scarce. Previous design methods for RNA–RNA switches are as follows. NUPACK [14,15] optimizes RNA sequences based on ensemble defects and hierarchical ensemble decomposition of the target secondary structures. To design RNA–RNA switch sequences that correspond to a specified reaction pathway, NUPACK models the reaction pathway with a set of “test tubes”. By de-optimizing off-target interactions between designed RNA sequences, NUPACK prevents the formation of undesired RNA complexes. RiboMaker [16] searches for a desired RNA sequence that optimizes the objective function (OF) values of the RNA–RNA switch design. RiboMaker adopts a heuristic approach for sequence optimization—simulated annealing. These previous tools are based on pseudoknot-free RNA structure prediction methods (e.g., RNAcofold in the Vienna RNA Package [17]); therefore, they are not suitable for designing joint secondary structures with an external pseudoknot, which frequently appear in natural non-coding RNA complexes, such as kissing hairpins. HyperFold can predict a general type of interacting RNA and has been applied to evaluate designed two-stranded RNA switches that output a small hairpin RNA in response to a prescribed input RNA sequence [18]. RCPred is a heuristic algorithm for predicting internal and external pseudoknots [19]. However, we could not find any design tools based on either HyperFold or RCPred as publicly available software.

These previous RNA–RNA switch design tools adopt mono-objective optimization techniques (i.e., they optimize a weighted sum of OFs). RNA–RNA switch design is a complex design problem and it requires more design objectives compared to those of typical single-sequence inverse folding. Since determining optimal weights between the objectives is a tedious task, it is desirable to use the framework of multi-objective optimization. By using multi-objective optimization, where our aim is to find out the Pareto-optimal solutions, we can optimize the RNA sequences without such empirical weights [20].

To design interacting RNAs with multi-objective optimization, we have extended MODENA [21] with RactIP [22,23], which can efficiently predict a general type of RNA joint secondary structure based on integer programming. MODENA+RactIP can design RNA–RNA switches whose joint secondary structures have an intermolecular kissing hairpin; moreover, within the framework of the integer programming, an interaction seed that plays a key role in initiating specific hybridization of RNAs is easily modeled and incorporated in the design of interacting RNAs. In addition to MODENA+RactIP, we extend MODENA with RNAcofold, which is included in the Vienna RNA package [17], to design RNA–RNA switches. We compared the design performance of these two design approaches (MODENA+RactIP and MODENA+RNAcofold) using a natural RNA–RNA switch dataset.

## 2. Algorithm

An RNA sequence is defined as a sequence of the nucleotides A, C, G, and U. Let us denote an RNA sequence $x_{\alpha }\in \Sigma^{n_{\alpha }}$, where Σ={A,C,G,U}, α is an index for RNA strands (i.e., α=1,2 for an RNA–RNA switch composed of two RNA strands), and $n_{\alpha }$ is the length of the strand α. A base pair is defined as a pair $\left(i_{\beta },j_{\sigma }\right)$ of nucleotide positions $i_{\beta }$ and $j_{\sigma }$ (β and σ indicate the strands), where AU, GC, and GU pairs are taken into account. For an intramolecular base pair, β=σ; for an intermolecular base pair, β≠σ. A secondary structure θ is a set of base pairs. If we explicitly deal with non-paired nucleotides (i.e., loop nucleotides), we can express the secondary structure as an array $b_{i}^{\alpha },(i=1,...n_{\alpha }$), where $b_{i}^{\alpha }$ has the position paired with position $i_{\alpha }$ if position $i_{\alpha }$ forms a base pair; otherwise, $b_{i}^{\alpha }$ contains a “.”, which indicates a loop nucleotide (i.e., an unpaired nucleotide). To address wild cards in the target secondary structures, we extend the definition of the target structure as ${\hat{b}}_{i}^{\alpha }$, in which *, +, @, and ⌃ can be specified to each nucleotide position; these letters mean “any structure is allowed”, “forming a base pair”, “forming an intramolecular base pair”, and “forming an intermolecular base pair”, respectively. The purpose of RNA inverse folding for RNA–RNA switch design is to optimize RNA sequences that fold into a specified extended joint target structure, ${\hat{b}}_{\alpha ,i_{\alpha }}^{\mathrm{hyb}}$ ($\alpha =1,2;i_{1}=1,...,n_{1};i_{2}=1,...,n_{2}$), and extended target structures, ${\hat{b}}_{j}^{1}$ ($j=1,...,n_{1}$) and ${\hat{b}}_{k}^{2}$ ($k=1,...,n_{2}$), where ${\hat{b}}_{\alpha ,i_{\alpha }}^{\mathrm{hyb}}$ is the array for a hybridized state.

A multi-objective optimization problem (MOP) addressed in the present study is defined as follows:

(1) $$\begin{array}{cc} \mathrm{minimize}\;\; & f_{i}\left(x_{1},x_{2}\right)\;\left(i=1,...,N_{f}\right),\\ \mathrm{subject}\;\mathrm{to}\;\; & g_{j}\left(x_{1},x_{2}\right)\leq 0\;\left(j=1,...,N_{g}\right),\\ \; & h_{k}\left(x_{1},x_{2}\right)=0\;\left(k=1,...,N_{h}\right),\\ \; & \left(x_{1},x_{2}\right)\in \{\left(s_{1},s_{2}\right)|s_{1}\in \Sigma^{n_{1}},s_{2}\in \Sigma^{n_{2}},\\ \; & \;\;\;\left(s_{1},s_{2}\right)\;\mathrm{is}\;\mathrm{compatible}\;\mathrm{with}\;\mathrm{the}\;\mathrm{set}\;\mathrm{of}\;\mathrm{target}\;\mathrm{structures}\}, \end{array}$$

where $g_{j}\left(x_{1},x_{2}\right)$ and $h_{k}\left(x_{1},x_{2}\right)$ are constraints; $N_{f}$ is the number of OFs, and $N_{g}$ and $N_{h}$ are those of the constraints (in the present study, $N_{h}$ = 0). An RNA sequence is said to be compatible with a target structure if any base pair in the target structure has the correct nucleotide assignment (e.g., AU, GC, or GU). The notion of compatibility is straightforwardly extended to the case of multiple sequences and multiple target structures.

The $f_{i}\left(x_{1},x_{2}\right)$, $g_{j}\left(x_{1},x_{2}\right)$, and $h_{k}\left(x_{1},x_{2}\right)$ are user-specified expressions that contain predicted values (free energy, structure distance, etc.) computed for a set ($x_{1}$ and $x_{2}$) of RNA strands obtained by the secondary structure prediction methods. To evaluate the generated interacting RNA sequences, RactIP or RNAcofold is invoked in MODENA. When predicting the structures of two isolated RNAs with RactIP, we utilize the structure constraint function of RactIP (“any intermolecular base pair formation is prohibited” is enforced for all nucleotide positions of the two RNAs). In the Vienna RNA Package, a prediction method (RNAfold) for a single sequence is used to predict the secondary structure of isolated RNAs.

### 2.1. Interaction Seed

**Figure 1:**
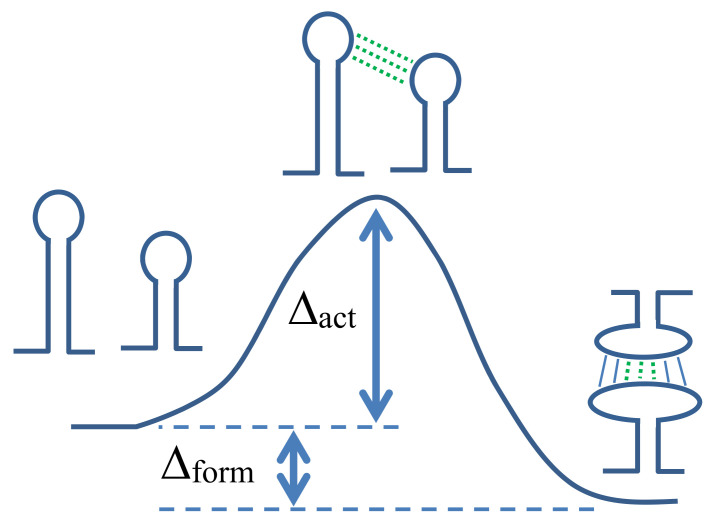
A schematic illustration of the hybridization process between two structured RNA strands. Two independently folded strands (shown on the left) are hybridized into the joint secondary structure (an RNA–RNA complex) shown on the right. Dotted lines are the base pairs of an interaction seed. Base pairs indicated by solid lines are intermolecular base pairs other than the base pairs of the interaction seed. $\Delta_{\mathrm{form}}$ and $\Delta_{\mathrm{act}}$ are the formation and activation free energy, respectively.

In RNA–RNA switch design, we consider the hybridization process between two structured RNA strands. A schematic illustration is shown in Figure 1. Since $\Delta_{\mathrm{act}}$ determines the transition rate between the two states, it is important to design RNA–RNA switches that have a small $\Delta_{\mathrm{act}}$ for the desired RNA–RNA complex formation. To include the transition state of the hybridization process into our RNA–RNA switch design, we adopt an approach that models the transition state with the formation of an interaction seed [16]. The interaction seed is a short helix composed of continuous intermolecular base pairs. When predicting the interaction seed, a short intermolecular helix with the largest hybridization probability or the lowest hybridization free energy is returned as a predicted interaction seed. In the present study, we assume that the interaction seed is a helix composed of continuous intermolecular base pairs, which are included in the predicted joint secondary structure (e.g., the continuous three base pairs depicted by dotted lines in Figure 1).

In RNA–RNA switch design, structure-constrained secondary structure prediction is useful for predicting an interaction seed between two structured RNA strands. We assume that the formation of a nascent complex between two RNA strands occurs through the formation of an interaction seed; therefore, the hybridization probability of the interaction seed should be maximized for desired RNA complexes and minimized for undesired ones (when we evaluate the interaction seed with free energy, the hybridization free energy should be minimized for desired RNA complexes and maximized for undesired ones) [16]. The integer programming framework and structure constraints of RactIP can straightforwardly deal with such an optimization. One of the structure constraints of RactIP can be specified for each nucleotide position. The structure constraints utilized to predict the interaction seed are as follows: A user-specified intramolecular base pair formation is enforced; any base pair formation is prohibited; any intramolecular base pair formation is prohibited.

By using RactIP, for a given maximum nucleotide length of the seed, we can find the optimal interaction seed between two RNA strands, each of which has an independently folded secondary structure. The interaction seed is searched for in a set of nucleotide positions named “seed candidates”; seed candidates are an intersection of the following two sets of nucleotide positions: (i) the nucleotides of the intermolecular base pairs in the predicted joint secondary structure; (ii) the loop nucleotides of the independently folded secondary structures.

**Figure 2:**
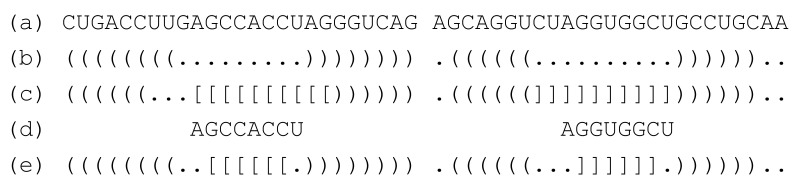
An example of the procedure for predicting an interaction seed. (**a**) Two RNA strands (strand 1 and strand 2). (**b**) Two independently predicted structures. (**c**) A predicted hybridized structure that has ten intermolecular base pairs. (**d**) Seed candidates (corresponding to the eight intermolecular base pairs); two intermolecular base pairs are not included in the seed candidates, since intramolecular base pairs are predicted at the same positions of strand 1 (see (**b**)). (**e**) A predicted toehold structure where the most stable interaction seed with a length of six nucleotides is predicted based on the seed candidates.

The procedure for obtaining an optimal interaction seed with RactIP is as follows: (i) First, fold two RNA strands independently (e.g., Figure 2b); this is achieved using the structure constraint of RactIP); then, obtain an optimal joint structure using RactIP (e.g., Figure 2c); (ii) constrain the independently folded secondary structures (other than the loop nucleotides belonging to the seed candidates) of the two RNA strands; the positions of the seed candidates (e.g., Figure 2d) are also constrained to avoid forming intramolecular base pairs, while the formation of intermolecular base pairs is allowed; (iii) run RactIP with the structure constraints mentioned in (ii) and the options such that “the number of accessible regions = 1” and “the maximum length of the accessible region = *w* nt” [23]. With these options, RactIP predicts one accessibility region at most, the maximum length of which is *w* nt (e.g., Figure 2e); this accessibility region corresponds to a single interaction seed.

To compute the interaction seed by using the Vienna RNA Package, we predict the lowest energy value of an interaction seed based on a small sliding window (with a length of *w* nucleotides) on each of the two strands. While scanning a predicted joint secondary structure, whenever the sliding windows find a pair of continuous seed candidates, the nucleotides of the sliding windows are folded using RNAcofold. After the scan is finished, the folded result with the lowest free energy in the scan is returned as the interaction seed.

### 2.2. Multi-Objective Optimization with Constraints

In general, we cannot expect that a single unique best solution exists for the MOP. Usually, the optimal solutions of MOPs are Pareto-optimal solutions due to the trade-offs between OFs. The MODENA web server provides an approximate set (i.e., multiple sets of two RNA sequences) of Pareto-optimal solutions based on a multi-objective genetic algorithm (GA). The optimal solutions against various weighted sums of OFs, which are obtained by using various weight values, are included in the Pareto-optimal solutions. By using a multi-objective optimization technique, we can perform RNA designs that take multiple OFs into account without empirical weights.

If the MOP has constraints, we have to handle the constraints properly in the optimization algorithm. In the present study, according to [20], we adopt the extended definition of dominance to include constraint violation in the comparison of two solutions: If solution A is feasible and solution B is not feasible, solution A dominates solution B; if both solution A and B are feasible, the standard definition of dominance is applied to these solutions, that is, if $f_{i}\left(A\right)$ is better than or equal to $f_{i}\left(B\right)$ for all *i*$\left(i=1,...,N_{f}\right)$ and $\exists j,f_{j}\left(A\right)\neq f_{j}\left(B\right)$, solution A dominates solution B; if both solution A and B are infeasible, that with less overall violation dominates the other one (when a tie in the overall violation occurs, neither solution dominates the other one). The overall violation of the constraints is calculated for each solution as $v\left(x_{1},x_{2}\right)=\sum_{i}^{N_{g}}\max\left(g_{i}\left(x_{1},x_{2}\right),0\right)+\sum_{i}^{N_{h}}\left|h_{i}\left(x_{1},x_{2}\right)\right|$.

### 2.3. An Overview of the MODENA Algorithm

Here, we briefly describe the algorithm of MODENA [21]. MODENA was developed based on the non-dominated sorting GA II (NSGA-II) [20]. In accordance with the standard GA, a population of solutions are generated in the initialization step; then, the evaluation step and reproduction step are iteratively performed until a stopping criterion is satisfied.

- The initialization step randomly generates $N_{p}$ solutions (sets of two RNA strands) that are compatible with the target structures, where $N_{p}$ is the population size. The $N_{p}$ solutions are set to a parent set *P* of solutions. A child set *C* of solutions is set to an empty set.
- In the evaluation step, prediction methods such as RactIP are invoked by using a system call to assign predicted structures and energies to the generated solutions. Then, all solutions in P+C are sorted using the non-dominated sorting [20], where less-dominated solutions are better solutions; if a tie occurs, that with a larger crowding distance [20] is considered as a better one. The top $N_{p}$ solutions are set to *P* and used as the parent solutions in the next reproduction step. The remaining worst $N_{p}$ solutions (if they exist) are deleted.
- In the reproduction step, the GA operators are called and applied to the parent solutions to generate $N_{p}$ child solutions. The tournament selection is used to select parent solution(s) to which one of the four GA operators is applied to generate one child solution. The set of the GA operators is comprised of point mutation for multiple targets, negative and positive design operators, and crossover for multiple targets. The point mutation selects nucleotide positions with a probability of $P_{M}$ to randomly change the nucleotides. The negative design operator assigns a pair of nucleotides that do not form a base pair to nucleotide positions, for which an undesired base pair is predicted. The positive design operator assigns a pair of G and C to the nucleotide positions to which a desired base pair specified in a target structure is not predicted. The crossover operator is applied to two parent solutions; each of these parent solutions is divided into two parts at a randomly selected nucleotide position; then, one child solution is constructed by concatenating the 5${}^{\prime }$ part of a parent solution and the 3${}^{\prime }$ part of another parent solution in a manner that does not destroy the compatibility with respect to the target structures. The generated child solutions are set to *C*.

As design functionalities, sequence constraints, in accordance with the International Union of Pure and Applied Chemistry (IUPAC) notation and mutation of undesired sequence motifs (these work as soft sequence constraints), are available in MODENA. Since the RNA sequence sampling algorithm adopted by MODENA takes the sequence constraints and the dependency graph into account, the generated RNA sequences rigorously satisfy the sequence constraints and are compatible with the target structures, even in cases when multiple target structures are specified. Mutation of undesired sequence motifs is performed just after the initialization and reproduction steps.

### 2.4. Objectives and Constraints for RNA–RNA Switch Design

In this design, we specify extended target structures of two RNA sequences and one joint extended target structure for when the two RNAs coexist and are hybridized. RNA sequences ($x_{1}$ and $x_{2}$) are designed to minimize the following three OFs: (i) the sum, $f_{\mathrm{dist}}$, of the structure distances for isolated ($d_{\mathrm{iso}}$) and hybridized ($d_{\mathrm{hyb}}$) states, where a structure distance is defined as the number of nucleotide positions to which an incorrect structure that is not consistent with the corresponding extended target secondary structure is predicted; (ii) the sum, $f_{\mathrm{int}}$, of interaction-related energies between the isolated and hybridized states, in which the free energy of an interaction seed ($E_{\mathrm{seed}}\left(x_{1},x_{2}\right)$) is minimized and the free energy difference ($\sum_{i\in \{1,2\}}E_{\mathrm{iso}}\left(x_{i}\right)-E_{\mathrm{hyb}}\left(x_{1},x_{2}\right))$ between the isolated and hybridized states is maximized; (iii) the free energy value $f_{E}$ for the hybridized state. The GC content is taken into account through constraints $g_{1}$ and $g_{2}$.

(2) $$\begin{array}{cc} f_{\mathrm{dist}}\left(x_{1},x_{2}\right) & =d_{\mathrm{hyb}}\left(x_{1},x_{2}\right)+\sum_{i\in \{1,2\}}d_{\mathrm{iso}}\left(x_{i}\right), \end{array}$$

(3) $$\begin{array}{cc} f_{\mathrm{int}}\left(x_{1},x_{2}\right) & =E_{\mathrm{seed}}\left(x_{1},x_{2}\right)+E_{\mathrm{hyb}}\left(x_{1},x_{2}\right)-\sum_{i\in \{1,2\}}E_{\mathrm{iso}}\left(x_{i}\right), \end{array}$$

(4) $$\begin{array}{cc} f_{E}\left(x_{1},x_{2}\right) & =E_{\mathrm{hyb}}\left(x_{1},x_{2}\right), \end{array}$$

(5) $$\begin{array}{cc} g_{1}\left(x_{1},x_{2}\right) & =c_{\mathrm{GC}}^{\inf}-c_{\mathrm{GC}}\left(x_{1},x_{2}\right), \end{array}$$

(6) $$\begin{array}{cc} g_{2}\left(x_{1},x_{2}\right) & =c_{\mathrm{GC}}\left(x_{1},x_{2}\right)-c_{\mathrm{GC}}^{\sup}, \end{array}$$

where the *E*s ($E_{\mathrm{iso}}\left(x_{i}\right)$, $E_{\mathrm{hyb}}\left(x_{1},x_{2}\right)$, and $E_{\mathrm{seed}}\left(x_{1},x_{2}\right)$) are the free energy values output by RactIP (for MODENA+RNAcofold, we use the minimum free energy (MFE) values as the *E*s). The structure distance of the isolated state is defined with the array representation of secondary structures as follows:

(7) $$\begin{array}{cc} d_{\mathrm{iso}}\left(x_{\alpha }\right) & =\sum_{i=1}^{n_{\alpha }}\delta_{i}^{\alpha }\\ \delta_{i}^{\alpha } & =\left\{\begin{array}{cc} 0 & \left(\mathrm{if}B_{i}\left(x_{\alpha }\right)\mathrm{is}\mathrm{consistent}\mathrm{with}{\hat{b}}_{i}^{\alpha }\right)\\ 1 & (\mathrm{otherwise}), \end{array}\right. \end{array}$$

where $B_{i}\left(x_{\alpha }\right)$ is the *i*-th element of the array representation of the secondary structure predicted for strand α, and ${\hat{b}}_{i}^{\alpha }$ is the *i*-th element of the target structure for strand α. The structure distance of the hybridized state is also defined as follows:

(8) $$\begin{array}{cc} d_{\mathrm{hyb}}\left(x_{1},x_{2}\right) & =\sum_{\alpha =1}^{2}\sum_{i=1}^{n_{\alpha }}\delta_{\alpha ,i}^{\mathrm{hyb}}\\ \delta_{\alpha ,i}^{\mathrm{hyb}} & =\left\{\begin{array}{cc} 0 & \left(\mathrm{if}B_{\alpha ,i}^{\mathrm{hyb}}\left(x_{1},x_{2}\right)\mathrm{is}\mathrm{consistent}\mathrm{with}{\hat{b}}_{\alpha ,i}^{\mathrm{hyb}}\right)\\ 1 & (\mathrm{otherwise}), \end{array}\right. \end{array}$$

where $B_{\alpha ,i}^{\mathrm{hyb}}\left(x_{1},x_{2}\right)$ and ${\hat{b}}_{\alpha ,i}^{\mathrm{hyb}}$ are the array elements for the hybridized state. User-prescribed $c_{\mathrm{GC}}^{\inf}$ and $c_{\mathrm{GC}}^{\sup}$ determine the allowed range of the GC content $c_{\mathrm{GC}}\left(x_{1},x_{2}\right)$ of the two RNA sequences. The maximum length *w* of the interaction seed is set to six base pairs [16].

In this set of OFs, to reduce runtime, we ignore destabilizing the interaction seeds of undesired homodimers (i.e., maximizing $E_{\mathrm{seed}}\left(x_{1},x_{1}\right)$ and $E_{\mathrm{seed}}\left(x_{2},x_{2}\right)$ is not included in the OFs mentioned above).

### 2.5. S+ Notation for Specifying Target Structures and Sequence Constraints

In the NUPACK: design web server, users can utilize DU+ notation [14] to specify the target secondary structures. In the MODENA web server, a notation (Shape-plus notation; in short, S+ notation) based on abstract shapes [24] and DU+ notation is available to specify the targets and sequence constraints. With this notation, by hand, users can easily edit the target secondary structures and sequence constraints.

**Figure 3:**
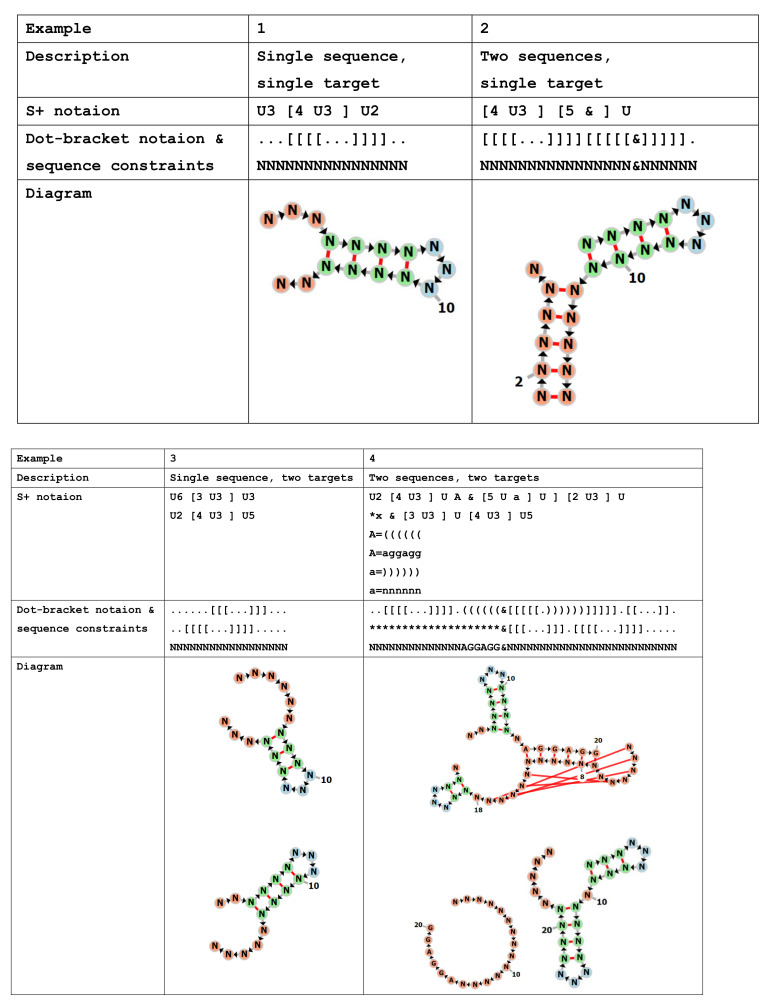
Toy examples of secondary structures specified in S+ notation. (Example **1**) A single RNA. (Example **2**) A complex of two RNAs. (Example **3**) A single RNA with meta-stable structures. (Example **4**) An RNA–RNA switch with sequence/structure motifs.

Toy examples of secondary structures specified in S+ notation are shown in Figure 3. Under the $\pi^{\prime }$ abstraction [24], an RNA secondary structure is represented by underbars and brackets (e.g., the shape of Example 1 in Figure 3 is _[_]_), where “_”, “[”, and “]” indicate loop nucleotides, upstream nucleotides of a duplex, and downstream nucleotides of a duplex, respectively. In S+ notation, inspired by DU+ notation, “U” can also be used to represent loop nucleotides. In the examples, the integer number followed by each of the “U” and “[” indicates the length of nucleotides (we can omit the integer number if the length is equal to one); the nucleotide lengths of duplexes are specified only for the upstream ones (i.e., “[”s), as with the DU+ notation described in the help page of the NUPACK: design website [14]. The symbol “&” is the boundary between the RNAs. The “x” that appears in Example 4 is a wild card for the integer number (this “x” is not available for the brackets); the value of the “x” is automatically determined in accordance with the length of another target structure. Capital and non-capital letters (except for “u”, “U”, “x”, and “X”) can be used to specify sequence motifs with their own secondary structure specifications, where a structure constraint and sequence constraint are assigned to each letter via two lines (e.g., in Example 4, A = “[[[[[[” and A = “aggagg”); the structure line is mandatory; if necessary, the sequence constraint line follows the structure line. The sequence constraint line can be omitted (if omitted, “N”, i.e., any nucleotide is acceptable, is assigned to each nucleotide position of the motif). In the secondary structures of the motif specification, the standard dot-bracket notation, only .[]()<>{} and their extension, *+@⌃, are allowed (integer numbers, “x”, and alphabets are not allowed in the motif specification).

### 2.6. Description on the Web Server

In the Supplementary Materials, we show screen shots of the MODENA web server. Figure S1 represents the top page of the web server. On the web page, users can select one of the design templates, including RNA–RNA switch design, designing two interacting RNAs, and inverse folding of a single RNA. In the submission page for the RNA–RNA switch design (Figure S2), there are two forms dedicated to the target structures in S+ notation and those in the standard bracket notation. Various parameters and prohibited motifs are also specified here. Figure S3 shows an example of RNA–RNA switch design using RactIP. In the designed results, the designed sequences and predicted structures are displayed. The predicted interaction seed and the result of elimination of prohibited motifs are also output. On the submission page and the result page, users can visualize the secondary structures using Forna [25]. The MODENA web server is available at http://rna.eit.hirosaki-u.ac.jp/modena/web (accessed on 8 March 2021).

## 3. Results and Discussion

To show the design performance of the web server, we benchmarked MODENA+RactIP with a dataset taken from a natural non-coding RNA complex dataset (the sample “RNA–RNA dataset” of RactIP 1.0.1). This dataset consists of eight sets of natural secondary structures (we call this dataset the natural dataset). The design results (mean values of $f_{\mathrm{dist}}$) obtained by performing five independent design runs for each target set are indicated in Table 1. For comparison, the design results for MODENA+RNAcofold, which cannot design the RNAs with joint secondary structures with an external pseudoknot, are also shown in the table. We used population sizes of 20, 50, 100, or 150, a maximum generation number of 150, $p_{M}$ = 0.03, $c_{\mathrm{GC}}^{\inf}$ = 45 (%), and $c_{\mathrm{GC}}^{\sup}$ = 55 (%). Homopolymer tracts ≥4 nt were prohibited. In these benchmark tests, we did not compare our tool with NUPACK and Ribomaker, since these two previous tools were not designed to accept external pseudoknots as input target structures. We found that MODENA+RactIP outperformed MODENA+RNAcofold on this dataset. This result is not surprising, since the target structures in the benchmark dataset contain secondary structures that belong to a class that cannot be predicted by RNAcofold. Although the design performance of MODENA+RactIP is high, there is still room for improvement, i.e., the $f_{\mathrm{dist}}$ value does not reach the perfect value (=0) yet. A possible reason for this result is the difficulty of the design targets. To test this hypothesis, we performed the RNA switch designs for a target set (we call this dataset the predicted dataset) that consisted of the target structures predicted by RactIP for the original RNA sequences of the benchmark dataset. As a result, with a population size of 150, for each of all the RNA switch species used in the benchmark, we obtained at least two sets of perfectly designed RNA sequences (i.e., $f_{\mathrm{dist}}$ = 0) whose predicted structures were exactly the same as the target ones. In Table 2, we show the number of successful runs in the five independent runs for each of the designs using the predicted dataset. Additionally, to further examine the design difficulty of the natural dataset, we performed designs that used a single target structure (only hybridized one) of the natural dataset, where MODENA+RactIP optimized the two OFs, $f_{\mathrm{dist}}^{\sin\mathrm{gle}}=d_{\mathrm{hyb}}\left(x_{1},x_{2}\right)$, and $f_{E}^{\sin\mathrm{gle}}=E_{\mathrm{hyb}}\left(x_{1},x_{2}\right)$. This design ought to be easier than that of RNA–RNA switch design, since the single-target design optimizes $d_{\mathrm{hyb}}\left(x_{1},x_{2}\right)$, while RNA–RNA switch design requires more targets (we optimized $d_{\mathrm{hyb}}\left(x_{1},x_{2}\right)+\sum_{i\in \{1,2\}}d_{\mathrm{iso}}\left(x_{i}\right)$). As a result, we still obtained values that did not reach the perfect value (= 0). One of possible reasons for this design difficulty is the prediction model adopted in the MODENA web server, where the maximum number of accessible regions is set to one, while two RNA–RNA interactions (CopA-CopT and OxyS-fhlA) in the natural dataset have external pseudoknots, thus requiring two accessible regions. This result of the single-target designs also indicates the difficulty of the interacting RNA designs with natural joint secondary structures with an external pseudoknot.

## 4. Conclusions

We developed a web server for designing molecular switches consisting of two interacting structured RNAs. Users can specify the secondary structures of two isolated RNAs and one hybridized state of the two RNAs as target structures, where we intend that these two RNAs will interact with each other and form a complex when these two RNAs coexist in vivo/vitro. In this web server, the secondary structures are predicted using RactIP or the Vienna RNA Package, and a set of two RNA sequences are optimized by a standard multi-objective genetic algorithm. The GC content of the RNA sequences is controlled through constraints during the design. The design performances were evaluated using the target secondary structures taken from the natural non-coding RNA complex dataset, where MODENA+RactIP showed high design performance for the benchmark target structures. Particularly, when we designed a set of RNAs that fold into the target structures predicted by RactIP, perfect design results were obtained for all target sets. To lower the energy barrier between two isolated RNAs, we considered an interaction seed that is modeled by the formation of an energetically stable intermolecular short helix. In the MODENA web server, with S+ notation, users can easily specify design targets, which include complicated base-paring information and sequence/structure motifs. Our web server is particularly useful if the user wants to design an RNA–RNA switch that has a joint secondary structure with an external pseudoknot, since the MODENA web server is currently the only web service that is capable of such designs.

## Figures and Tables

**Table 1:** Comparison of the design benchmark results for MODENA+RactIP and MODENA+RNAcofold. The mean structure distance for the dataset is shown, where only the lowest structure distance in each of the RNA species is taken into account. The “predicted dataset” indicates the designs with the target structures predicted by RactIP. The “single target” indicates the results for single-target joint structure designs, where the natural dataset was used. A smaller value indicates a better design.

Design Methods	Population Size			
	20	50	100	150
MODENA+RactIP (predicted targets)	6.8	2.4	0.9	0.5
MODENA+RactIP	17.9	13.4	12.3	12.0
MODENA+RNAcofold	37.8	30.5	28.1	27.1
MODENA+RactIP (single target)	11.5	10.7	10.3	10.0

**Table 2:** The number of “perfect design runs” in the five independent runs (with different initial random numbers) of “MODENA+RactIP (predicted dataset)”.

RNA Pair	Population Size			
	20	50	100	150
CopA-CopT	4	5	5	5
DIS-DIS	3	5	5	5
IncRNA54-RepZ	5	5	5	5
MicA-ompA	0	0	4	4
OxyS-fhlA	0	1	2	4
R1inv-R2inv	5	5	5	5
RyhB-SodB	0	0	0	2
Tar-Tarstar	5	5	5	5

## Data Availability

The target structures used in the benchmark are available on the MODENA web server.

## Supplementary Materials

The following are available online at https://www.mdpi.com/1422-0067/22/5/2720/s1, Figure S1: The top page of the MODENA web server; Figure S2: The submission page for RNA–RNA switch design on the MODENA web server; Figure S3: An example of the result page output from the MODENA web server.

## Abbreviations

The following abbreviations are used in this manuscript:

RNA	Ribonucleic Acid
STAR	Small Transcription-Activating RNA
UTR	Untranslated Region
RBS	Ribosome Binding Site
CRISPR	Clustered Regularly Interspaced Short Palindromic Repeat
sgRNA	small guide RNA
OF	Objective Function
A	Adenine
C	Cytosine
G	Guanine
U	Uracil
MOP	Multi-objective Optimization Problem
GA	Genetic Algorithm
IUPAC	International Union of Pure and Applied Chemistry
MFE	Minimum Free Energy
