# Supplementary material for: A Web Server for Designing Molecular Switches Composed of Two Interacting RNAs
Source: Int J Mol Sci. 2021 Mar 8;22(5):2720. doi: 10.3390/ijms22052720 (PMC7962656; doi:10.3390/ijms22052720)
Supplement: Supplementary file 1 [file ijms-22-02720-s001.pdf]

Supplementary Materials for

“A web server for designing molecular switches composed of two interacting RNAs”

Akito Taneda and Kengo Sato

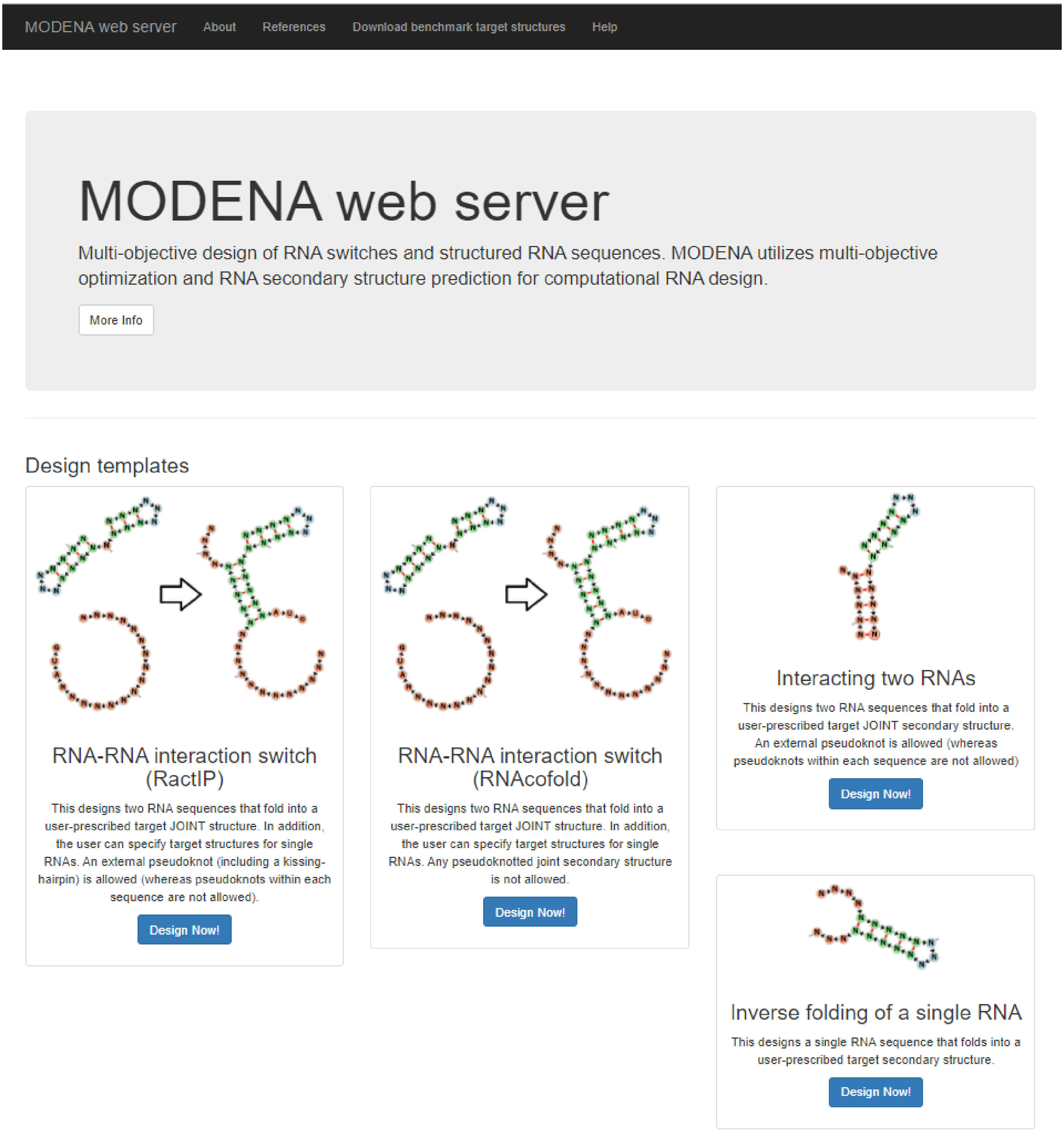

Figure S1. The top page of MODENA web server.

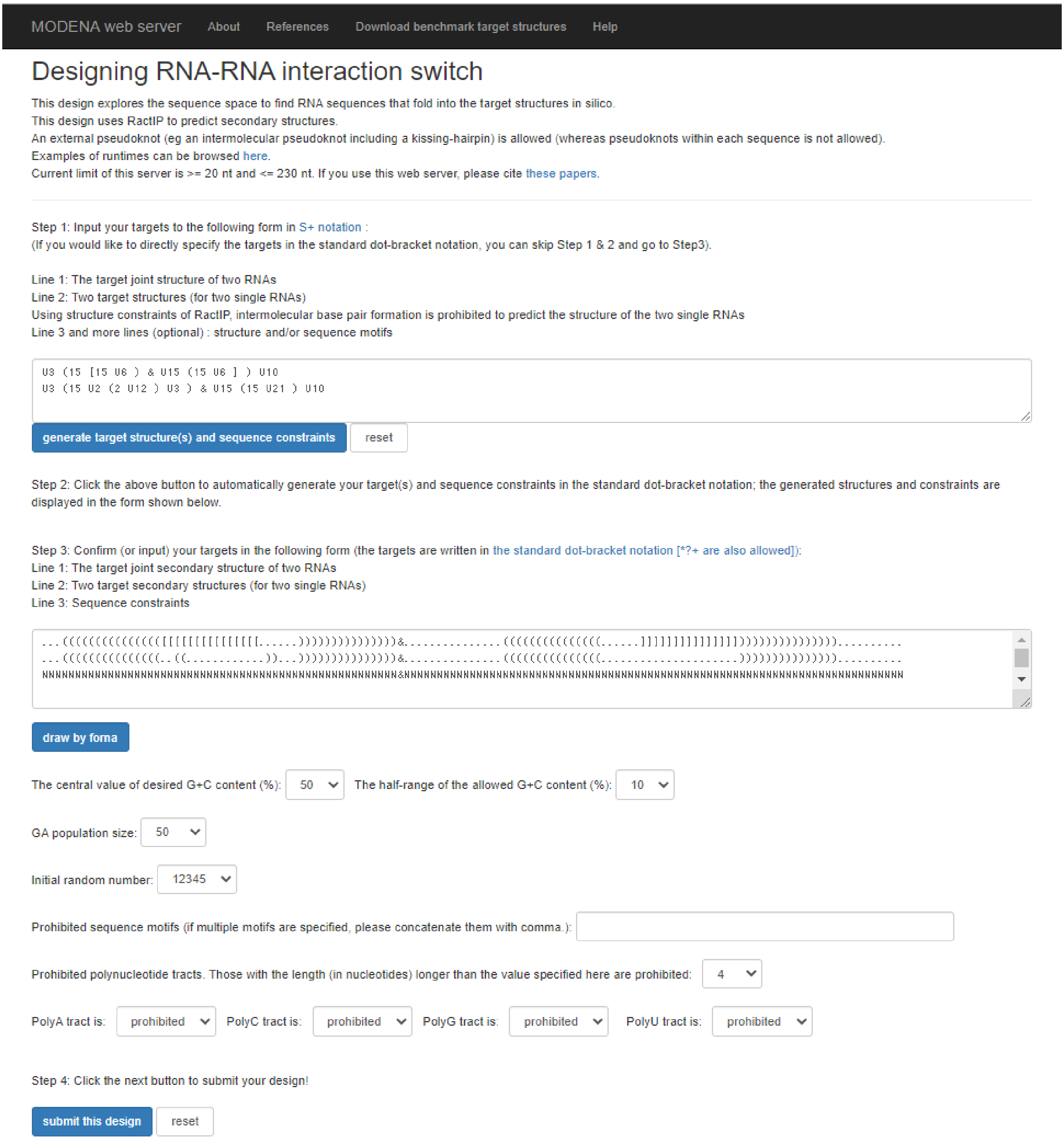

MODENA web server | About | References | Download benchmark target structures | Help

Designing RNA-RNA interaction switch

This design explores the sequence space to find RNA sequences that fold into the target structures in silico.
This design uses RactIP to predict secondary structures.
An external pseudoknot (eg an intermolecular pseudoknot including a kissing-hairpin) is allowed (whereas pseudoknots within each sequence is not allowed).
Examples of runtimes can be browsed here.
Current limit of this server is >= 20 nt and <= 230 nt. If you use this web server, please cite these papers.

Step 1: Input your targets to the following form in S+ notation :
(If you would like to directly specify the targets in the standard dot-bracket notation, you can skip Step 1 & 2 and go to Step3).

Line 1: The target joint structure of two RNAs
Line 2: Two target structures (for two single RNAs)
Using structure constraints of RactIP, intermolecular base pair formation is prohibited to predict the structure of the two single RNAs
Line 3 and more lines (optional) : structure and/or sequence motifs

```
U3 (15 [15 U6 ) & U15 (15 U6 ] ) U10
U3 (15 U2 (2 U12 ) U3 ) & U15 (15 U21 ) U10
```

generate target structure(s) and sequence constraints | reset

Step 2: Click the above button to automatically generate your target(s) and sequence constraints in the standard dot-bracket notation; the generated structures and constraints are displayed in the form shown below.

Step 3: Confirm (or input) your targets in the following form (the targets are written in the standard dot-bracket notation [*?+ are also allowed]):
Line 1: The target joint secondary structure of two RNAs
Line 2: Two target secondary structures (for two single RNAs)
Line 3: Sequence constraints

```
...((((((((((((((([[[[[[[[[[[[[[[......)))))))))))))))&...............(((((((((((((((......]]]]]]]]]]]]]]])))))))))))))))..........
...(((((((((((((((..((............))...)))))))))))))))&...............(((((((((((((((.....................)))))))))))))))..........
NNNNNNNNNNNNNNNNNNNNNNNNNNNNNNNNNNNNNNNNNNNNNNNNNNNNNN&NNNNNNNNNNNNNNNNNNNNNNNNNNNNNNNNNNNNNNNNNNNNNNNNNNNNNNNNNNNNNNNNNNNNNNNNNNNNNNNN
```

draw by forna

The central value of desired G+C content (%): 50 The half-range of the allowed G+C content (%): 10

GA population size: 50

Initial random number: 12345

Prohibited sequence motifs (if multiple motifs are specified, please concatenate them with comma.):

Prohibited polynucleotide tracts. Those with the length (in nucleotides) longer than the value specified here are prohibited: 4

PolyA tract is: prohibited PolyC tract is: prohibited PolyG tract is: prohibited PolyU tract is: prohibited

Step 4: Click the next button to submit your design!

submit this design | reset

Figure S2. The submission page of RNA-RNA switch design in MODENA web server.

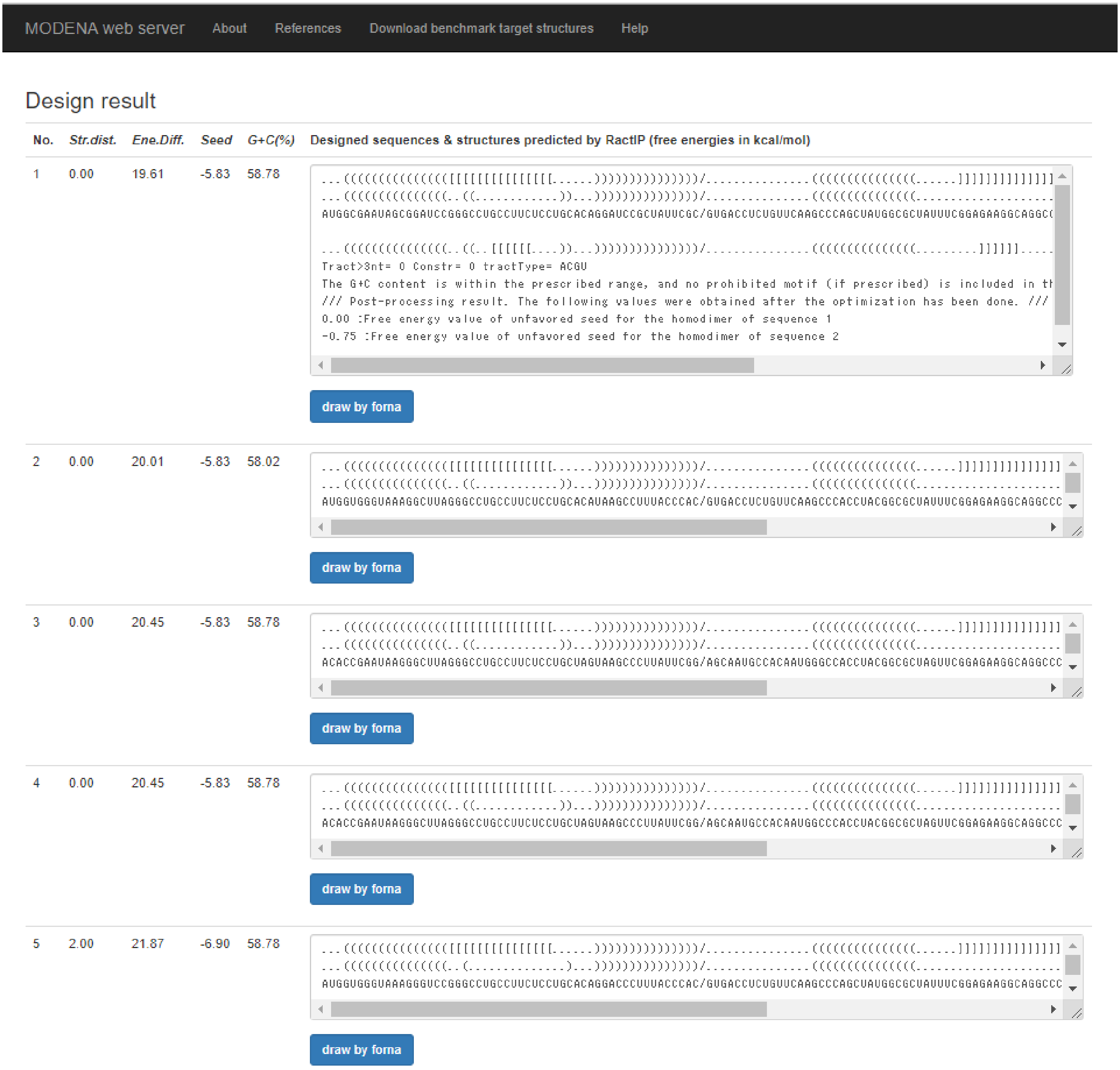

Figure S3. An example of the result page output from MODENA web server.
